# Violence exposure and quality of life among partners and parents of individuals with substance use problems

**DOI:** 10.3389/fpubh.2026.1863383

**Published:** 2026-09-11

**Authors:** Bente Birkeland, John-Kåre Vederhus, Anne Schanche Selbekk, Anne-Marie Laslett, Ingrid Mary Wilson, Siri Håvås Haugland

**Affiliations:** 1 Department of Psychosocial Health, University of Agder, Grimstad, Norway; 2 Sørlandet Hospital, Children's Best Interest (Barns Beste), Kristiansand, Norway; 3 Addiction Unit, Sorlandet Hospital, Kristiansand, Norway; 4 Department of Public Health, University of Stavanger, Stavanger, Norway; 5 Center for Alcohol and Drug Research (KORFOR), Stavanger University Hospital, Stavanger, Norway; 6 Center for Alcohol Policy Research, La Trobe University, Melbourne, VIC, Australia; 7 Care Economy Research Institute, La Trobe University, Melbourne, VIC, Australia; 8 Singapore Institute of Technology, Singapore, Singapore; 9 Judith Lumley Center, La Trobe University, Melbourne, VIC, Australia

**Keywords:** affected family members, gender, quality of life, substance use disorders, violence

## Abstract

**Background:**

Substance use problems (SUP) have significant adverse consequences not only for individuals with such problems, but also for their loved ones. One important concern is exposure to violence among affected family members. This study investigates whether close family members, specifically partners or parents, of individuals with SUP, including alcohol, face increased risks of physical and sexual violence and emotional abuse within the last 5 years. We also examined a hypothesized mediation model linking SUP exposure, violence, and quality of life (QoL).

**Methods:**

We conducted a cross-sectional analysis using data from 17,640 adults aged 23 years and above who participated in the Norwegian Counties Public Health Survey 2023. Multivariable logistic regressions were performed, controlling for age and socioeconomic status, and stratified by gender. Latent regression analysis was used to examine the relationship between SUP exposure and QoL.

**Results:**

Gender-specific analyses revealed that partners or parents of individuals with SUP had significantly higher odds of experiencing physical violence (women OR 4.54; men OR 4.14), sexual violence (women OR 4.31; men OR 4.80), and emotional abuse (OR 2.96 across genders). These family members also reported lower QoL. The association between family member status and lower QoL was statistically accounted for by experiences of violence. Information on the perpetrator was not available, and the source of reported violence could therefore not be determined.

**Conclusion:**

Close family members such as partners or parents of individuals with SUP face substantial risks of violence and emotional harm and report lower QoL. Support services should offer gender-sensitive, tailored assistance that addresses family members' needs as individuals, not solely as caregivers. These findings highlight the importance of recognizing affected family members as a vulnerable population within public health initiatives.

## 1 Introduction

Substance use problems (SUP), including alcohol, represent a considerable public health challenge worldwide (1). However, problems related to excessive use of alcohol and drugs are not limited to the person using them. Problematic use can affect family members and close relations in profound ways, both across generations and within the same generation, and impact parents, partners, children, siblings, or other significant others (2–5).

The prevalence of close family members affected by another person's SUP varies by location and substance, reflecting differences in SUP prevalence (6, 7). Research shows challenges in estimating the impact of alcohol's harm to others (8) and multi-country estimates of women affected by a partner's drinking vary widely, with negative effects reported by 4% of women in Nigeria and the USA up to 33% in Vietnam (9). In Australia, 18.6% of women reported harm from the alcohol use of a household member, intimate partner, or relative, while the prevalence among men was 10% (2). Estimates in Norway have shown that many people live with a person who uses alcohol at high-risk levels: 50–100,000 Norwegians reported that they lived with a partner who's alcohol use had an impact, representing 1–2% of the adult population, and 100–250,000 reported living with another (unspecified) family member who drank riskily, representing 2–6% of the adult population (10). The wide variation and uncertainty in existing estimates suggests that the topic has not received sufficient attention. This limited focus on close family members within SUP treatment is evident in both research and clinical practice. Family members are mainly involved in treatment trajectories to provide valuable resources for the person with SUP (11), and their own needs for support as impacted family members are often overlooked (12), as are the consequences for the family as a whole (13).

When someone struggles with SUP, it can have far-reaching effects on close family members (14, 15) and negatively affect both the physical and psychological well-being of these family members (16–19). SUP is experienced as intrusive and disturbing to the dynamics and relations within a family and can be harmful to overall family functioning (20–22). A serious concern regarding the potential adverse outcomes of SUP is that it is associated with various forms of family violence, such as verbal aggression, physical violence, and physical or verbal abuse within the family (23–28). Family violence can take different forms, such as intimate partner violence (IPV), domestic violence (24), or violence from children toward parents (29). When alcohol is involved in family violence, the violence is more severe (30), resulting in more significant injuries, and associated with increased risk of fatal outcomes (31, 32).

Studies of partners of people with SUP have found strong associations between SUP and violence, poor physical and mental health, and poor overall quality of life (QoL) (33, 34). Partners in such situations qualitatively describe everyday lives that include threats from their partner, constant fear for both their own life and their partner's life, and worry about how to protect their children in these situations (35). According to a recent systematic review and modeling study (36) across 18 European countries, the pooled prevalence of partner-related violence associated with drinking was 0.6% for physical violence, 0.1–1.6% for sexual violence, and 2.7% for emotional violence. Wilson et al (3) summarized various types of alcohol-related harms women experience, including aggression and violence, sexual coercion and violence, economic abuse, and controlling behaviors. Ólafsdóttir et al. (37) conducted interviews with family members (spouses, partners, siblings, and children) of individuals with a substance use disorder. Their findings revealed that in addition to experiences of physical abuse, all participants reported experiencing emotional abuse.

Although some studies have shown that adolescents' SUP have a negative impact on families‘ QoL and functioning (38, 39), few studies have investigated the experiences and challenges encountered by parents of adolescents with substance use issues (27, 40). According to a study by Usher et al. (40), parents of adolescents with SUP quite often experience both physical and verbal abuse. This was confirmed in another study by Hoeck and Van Hal (27), who found that parents of individuals with SUP commonly experienced mental abuse, including verbal abuse, rudeness, domineering behavior, and threats. Additionally, physical violence, such as pushing, punching, and hitting, was also prevalent.

Taken together, partners and parents of people with SUP experience high levels of stress, health problems, and social strain. These challenges are not only private family matters but also an important public health concern. Because many families are affected, the impact extends beyond individual households and places a broader burden on health and social services. Acknowledging the health of relatives as part of the public health picture is therefore important for prevention, early support, and long-term population wellbeing.

There is a need for more attention to and action on family aggression and violence in the context of SUP (26, 41, 42). From a Norwegian context, few studies have explored self-reported family violence and abuse among family members of persons with SUP (36). According to a proposition to the Norwegian Parliament (43), expertise on violence in close relationships should inform health, family, and care services. Yet, according to a larger American study, only a minority of treatment facilitations for SUP include services related to violence (41). Thus, knowledge about all forms of violence is crucial to screening and tailoring effective interventions, for the person using substances, for family members, and for families as a whole (23, 44, 45).

Utilizing data from a broad age range in a general population in Norway, this study first examines the prevalence of being a partner or parent to an individual with SUP, as well as recent experiences of physical violence, sexual violence, and emotional abuse. It then investigates whether these family members are at increased risk of such forms of violence, with particular attention to gender-specific risk patterns. Additionally, we examine a hypothesized mediation model linking family exposure, violence, and QoL.

## 2 Methods

### 2.1 Participants and procedures

Drawing on the Agder public health study, conducted in Norway's southernmost county in collaboration with the Norwegian Institute of Public Health (NIPH) and Agder County Council (46), adults born before September 1, 2005 who were “ordinary residents” with a valid permanent national ID number and an ordinary living address (e.g., not commuter status and not a client of an institution) were eligible for sampling (*n* = 217,785). A representative sample of 57,891 individuals was drawn from this pool. Data collection was undertaken through a digital survey and took place from September 20 to October 23, 2023. A total of four reminders were sent: two by email and two by SMS (46). Of the total number of individuals invited, 18,517 chose to participate in the study, resulting in a response rate of 32%. The current study included individuals aged 23 years or older due to the focus on adverse life events occurring in adulthood within the past 5 years (*n* = 17,640). Following ethical approval, the NIPH, which handled the data on a secure server, made it available for analysis to a project group at the University of Agder.

### 2.3 Measures

Age and gender were obtained from the Central Population Register. Participants (*n* = 144) older than 84 years were coded as 85 for anonymity reasons.

#### 2.3.1 Close relative of person with alcohol or SUP

Respondents were asked whether they, after reaching the age of 18 years, had experienced or were currently experiencing being a partner or parent to an individual with SUP or substance use disorder, based on their own assessment rather than formal diagnosis. It is relevant to note that the Norwegian term for 'substance use problems' encompasses both alcohol-related problems and problems related to other substances. The response options were yes/no.

#### 2.3.2 Experience of violence in adulthood (last 5 years)

Respondents were asked to report whether they had experienced any of the three types of violence outlined within the past 5 years. These events included physical violence, being forced or being subject to attempts to be forced into sexual intercourse, and prolonged experiences of humiliation or degradation. For each event, respondents were provided with response options of “Yes” and "No”. In this paper, the variables representing these events are referred to as “physical violence”, “sexual violence”, and “emotional abuse”. These indicators were subsequently modeled as reflecting a common latent construct of violence to capture their shared underlying dimension while accounting for measurement error.

#### 2.3.3 QoL

QoL was measured using the Essential QoL-3 (EQoL-3) scale, which is an ultra-short instrument assessing core QoL constructs. The scale includes three items assessing life satisfaction, sense of meaningfulness in life, and emotional wellbeing (happiness). Each item was scored on a 0–10 point scale, with higher scores indicating greater satisfaction, happiness, and perceived meaningfulness. The scale demonstrated good internal consistency in the validation study, with a Cronbach's alpha of 0.86 (47).

#### 2.3.4 Low socioeconomic status

The variable “financial difficulties” served as a proxy for low socioeconomic status (SES). It aimed to measure the level of ease or difficulty individuals faced in meeting their day-to-day needs, accounting for their income. For single-person households, respondents were asked to consider their total income, while participants from households with multiple members were asked to consider the household's income. Respondents rated their situation on a 7-point scale: “very easy”, “easy”, “somewhat easy”, “somewhat difficult”, “difficult”, and “very difficult”. For analysis, the variable was dichotomized, with responses from “somewhat difficult” to “very difficult” categorized as indicative of low SES.

### 2.4 Statistical analysis

Descriptive analyses were conducted to provide an overview of the material as well as crude associations. Applying gender-stratified multivariable logistic regression, we compared the odds of life adversities of violence in adulthood between those who identified themselves as a partner or parent to someone with SUP and individuals without this family exposure. Associations were reported as odds ratios (OR) with 95% confidence intervals (CI) and adjusted for age and SES. These analyses were conducted using IBM SPSS Statistics, version 30 (IBM Corp., Armonk, NY). In a simultaneous model, a structural equation modeling (SEM) regression analysis examined the association between being a partner or parent to an individual with SUP, experiencing violence in the last 5 years, and QoL. Violence and QoL were specified as latent variables, allowing estimation of direct and indirect (mediated) effects of this family exposure on QoL. The model was adjusted for age, gender, and SES. Given the categorical nature of the violence indicators, we used the weighted least squares mean and variance adjusted (WLSMV) estimator, which uses all available data under a missing-at-random assumption. Results are reported as unstandardized β coefficients to facilitate interpretation, along with R^2^ values indicating the proportion of variance in QoL explained by the model. The model fit was evaluated using root mean square error of approximation (good fit ≤ 0.06; acceptable ≤ 0.08) and comparative fit index (good fit ≥0.95; acceptable ≥0.90) (48). Analyses were conducted in Mplus version 8.6 (Muthén & Muthén, Los Angeles, CA).

## 3 Results

The study included 9,740 women (55.2%) and 7,900 men (44.8%), with a mean age of 53.1 years (standard deviation: 15.3). One in four reported financial difficulties (25.7%), indicating low SES.

A somewhat higher proportion of women identified themselves as partners or parents of someone with SUP (17.8% vs. 15.0%; *p* < 0.001). The prevalence of all three forms of violence was higher among women than among men. The difference was particularly pronounced for sexual violence (3.3% among women vs. 0.7% among men) and emotional abuse (8.1% among women vs. 5.1% among men; Table 1). A more detailed analysis of missingness is provided in Supplementary Table 1.

**Table 1:** Descriptive statistics, Norwegian Counties Public Health Survey (Agder 2023).

Variables	Women		Men		*p*-value	Missing
	*n*	%	*n*	%		*N*
Partners or parents of individuals with SUP	1,719	17.8	1,177	15.0	*P* < 0.001	114
Physical violence in the last 5 years	322	3.4	200	2.6	*P* = 0.002	1,137
Sexual violence in the last 5 years	315	3.3	54	0.7	*P* < 0.001	1,162
Emotional abuse in the last 5 years	774	8.1	396	5.1	*P* < 0.001	1,193

*N* = 17,640.

**Figure 1:**
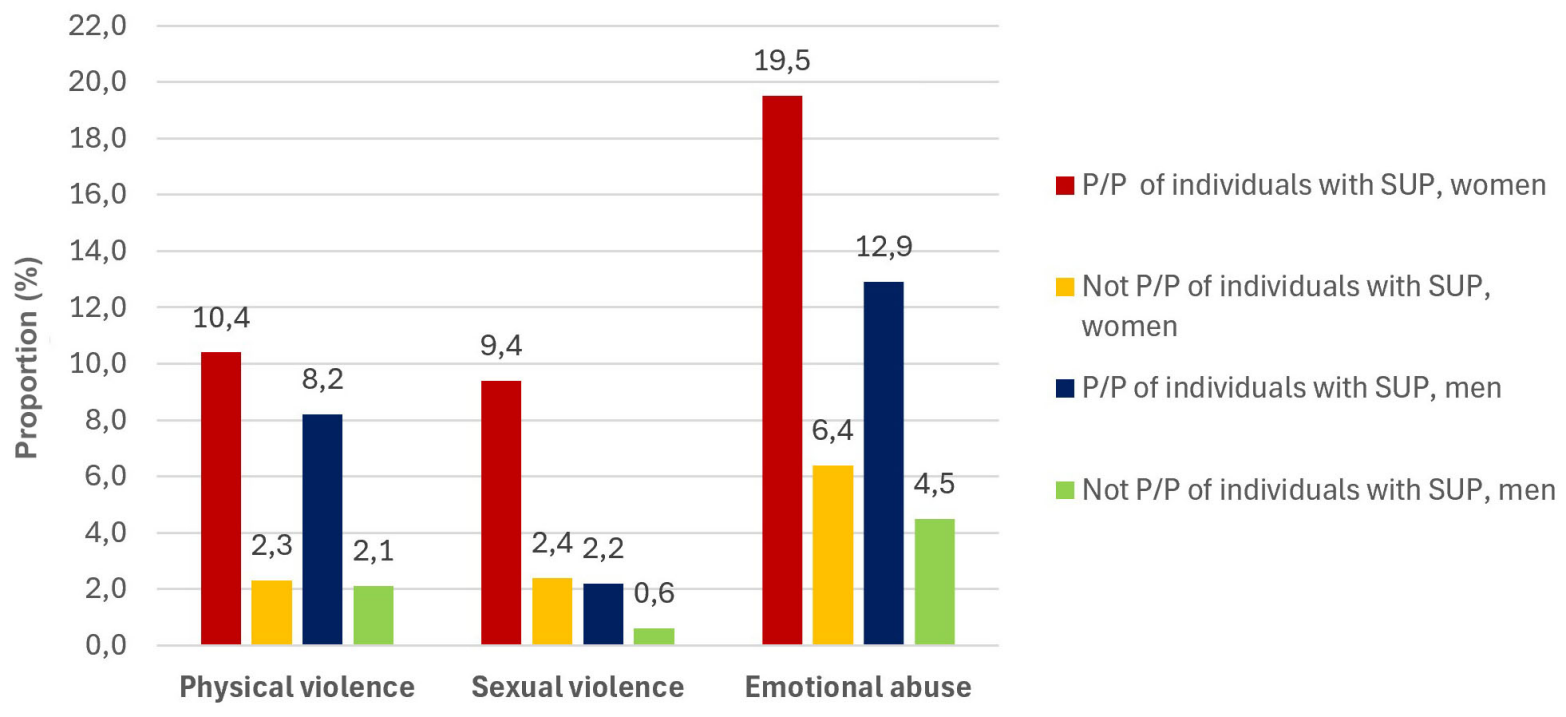
Prevalence of violence exposure^a^ among partner/parents (P/P) of individuals with Substance use problems (SUP) vs others, by gender. ^a^Last 5 years, *N* = 17,640.

Figure 1 shows that all types of violence were more prevalent among women and men who were partners or parents of individuals with SUP compared to participants who were not. Among these family members, 10.4% of women and 8.2% of men experienced physical violence in the past 5 years. Within each gender, these rates were approximately four times higher than among individuals without SUP in their family. The prevalence of sexual violence was also notably higher among these close family members, with 9.4% of women and 2.2% of men reporting such experiences. Both rates of sexual violence were approximately four times higher than those among individuals without SUP in their family. Emotional abuse was also more common among the partners or parents of someone with SUP, with reporting by one in five women and one in eight men.

Table 2 shows that, after adjusting for age and SES in multivariable analyses, both female and male partners or parents of individuals with SUP had strongly increased odds of experiencing violence in the last 5 years compared to individuals without this family exposure. In these gender-stratified analyses, comparing partners or parents of someone with SUP to others of the same gender without this family exposure, partners or parents had fourfold or higher odds of physical and sexual violence. The odds of emotional abuse were nearly three times higher across genders.

**Table 2:** Associations between partner/parent of individuals with SUP and adulthood violence exposure, stratified by gender.

Variables	WOMEN			MEN		
	Physical violence	Sexual violence	Emotional abuse	Physical violence	Sexual violence	Emotional abuse
Partner or parent of individual with SUP (crude)	4.90 (3.88–6.19)^***^	4.23 (3.33–5.37)^***^	3.51 (2.97–4.15)^***^	4.19 (2.99–5.87)^***^	3.98 (2.12–7.47)^***^	3.17 (2.42–4.15)^***^
Partner or parent of individual with SUP (adjusted^2^)	4.54 (3.55–5.81)^***^	4.31 (3.33–5.58)^***^	2.96 (2.54–3.61)^***^	4.14 (2.89–5.92)^***^	4.80 (2.48–9.31)^***^	2.96 (2.23–3.94)^***^

SUP, Substance use problems. Reference group: individuals without such family exposure. Results from multivariable logistic regression are adjusted for age and SES and presented as odds ratios with 95% confidence intervals.

**Figure 2:**
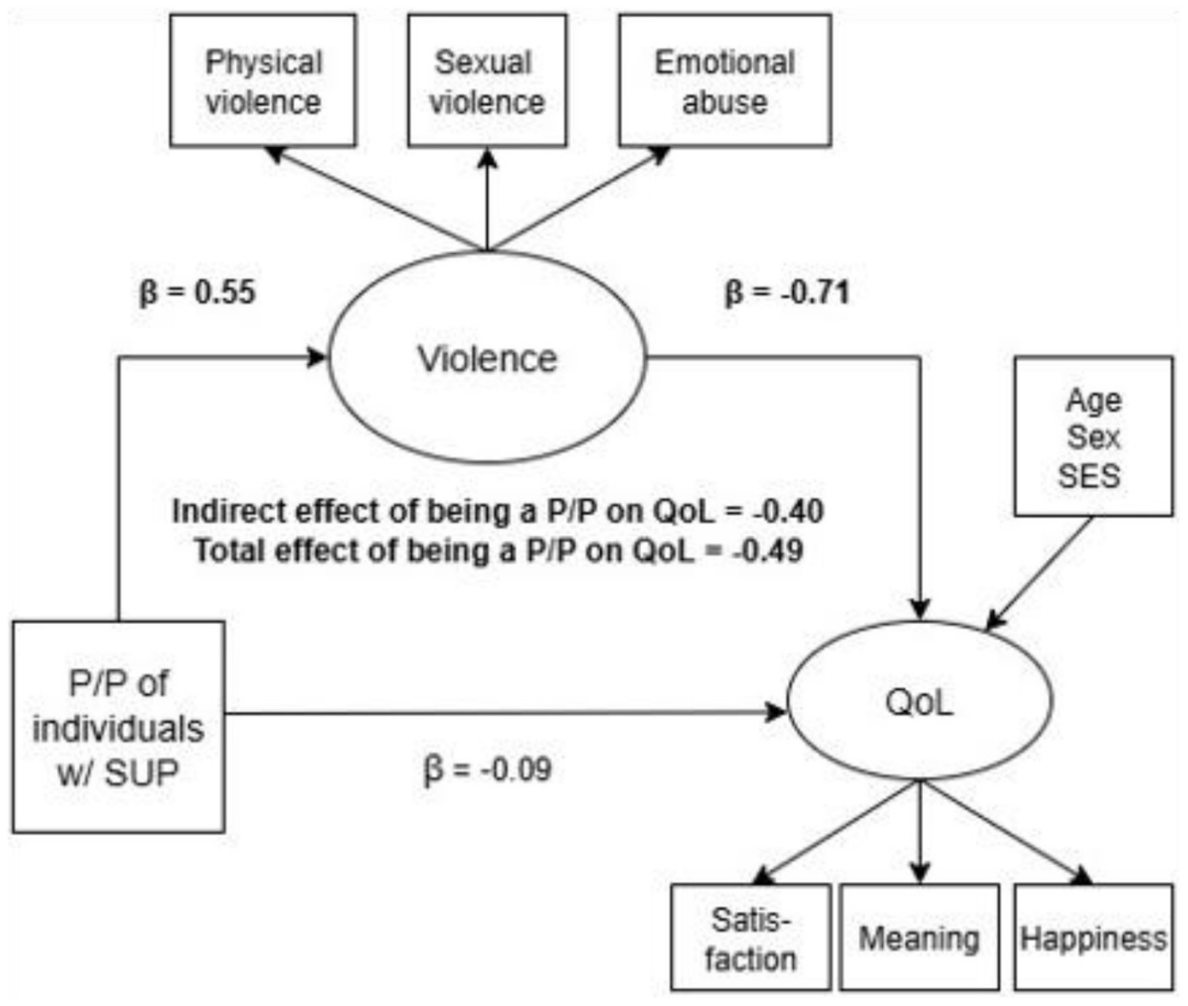
Latent regression analysis of QoL linked to violence exposure and partner/parent (P/P) status of individuals with, Substance use problems (SUP) exposure. Unstandardized beta coefficients (β) between the structural elements are presented, including the estimated indirect effect within the hypothesized mediation model. Coefficients in bold indicate statistical significance at the *p* < 0.001 level, while non-bold coefficients did not reach the *p* < 0.05 threshold. The results are controlled for age, gender, and socio-economic status (SES).

As in the logistic regression analyses, the SEM analysis (Figure 2) revealed that being a partner or parent to someone with SUP was significantly associated with increased exposure to violence in the past 5 years (β = 0.55; 95% CI = 0.48–0.63; *p* < 0.001). In turn, exposure to violence was strongly associated with lower QoL (β = −0.71; 95% CI = −0.80/−0.63; *p* < 0.001). The estimated indirect effect of family exposure on QoL through violence was β = −0.40 (95% CI = −0.46/−0.33; *p* < 0.001). The estimated direct effect of family exposure on QoL was β = −0.09 (*p* = 0.073), resulting in a total effect of β = −0.49 (95% CI = −0.57/−0.40; *p* < 0.001). As the direct association was non-significant, the observed association between this family exposure and QoL was statistically accounted for by experiences of violence. The model accounted for 23% of the variance in QoL (*R*^2^ = 0.23).

## 4 Discussion

In this study, which utilized a large sample from the general population, we found that partners or parents of individuals with SUP had a strongly increased risk of experiencing violence in the last 5 years compared to individuals without this family exposure. The odds within each gender were generally comparable. The association between being a partner or parent to someone with SUP and reduced QoL was statistically accounted for by exposure to violence.

Partners or parents of individuals with SUP were at substantially higher risk of experiencing physical violence, sexual violence, and emotional abuse over the past 5 years compared with other participants. Regarding partners, this finding is in line with previous studies (23, 25), which show that heavy drinking is significantly associated with partner abuse. According to Foran and O'Leary (24), certain forms of drinking, such as binge or heavy drinking, are more associated with aggression. These findings extend to other substances as well. Stalans and Ritchie (49) demonstrated that the risk of IPV is significantly associated with other forms of substance use as well. The findings also align with the qualitative study by Hoeck and van Haal (27), showing that violence, including aggression, verbal abuse, threats, and physical assault, was commonly experienced by parents. A qualitative study highlighting a cycle of drinking and violence showed women experience heightened aggression from a heavy drinking partner when that partner is highly intoxicated and that challenging or confronting the partner increased the risk of violence (35). These patterns of elevated violence exposure among individuals living with a family member who uses substances, as described above, reflect general trends observed across different studies. However, the violence-related items included in the present study did not allow us to identify the perpetrator. Thus, with the available data, we were not able to establish with certainty whether the violence was perpetrated by the family member with SUP.

In addition to humiliation and violence, previous studies have reported substantial stigma and shame associated with being affected by SUP within a family, and, for various reasons, fear of confronting the family member about their alcohol use (3, 50). All these factors may contribute to raising the threshold for seeking help or support. However, although studies suggest that peer support groups can be helpful, the threshold for seeking such support remains high (27, 50, 51). Many close family members of individuals with SUP thus live in isolated situations with a high risk of serious substance use-related consequences from their family member's actions.

Although women had a higher absolute prevalence of victimization than men across all three types of violence, gender-stratified analyses showed that, within each gender, being a partner or parent to someone with SUP was associated with roughly similar increased odds of experiencing physical violence, sexual violence, and emotional abuse compared to those who were not partners or parents within the same gender. For women, this is in line with previous research, showing that 14–40% of women experience harm from relatives who drink, most often from men with whom they are in close relationships (52). Men with high levels of alcohol consumption have a greater tendency to perpetrate IPV (52–54). It is encouraging that a 2023 Norwegian governmental knowledge synthesis on women's health addressed IPV and acknowledged the link between substance use and violence. The report recommended developing targeted interventions for women in alcohol and drug treatment (55).

Although much of the literature emphasizes women as the primary victims of sexual violence, our analyses conducted separately for each gender showed that male partners or parents of individuals with SUP also face a higher risk of sexual violence compared to men with no such experience of SUP. It is possible that societal stigma and gender norms may contribute to underreporting among men. Our findings highlight the need for gender-inclusive research and trauma-informed support systems that recognize the diverse experiences of all family members affected by SUP. Across substance use treatment settings, the implementation of a gender-responsive approach that explicitly addresses substance use-related violence may be beneficial. Overall, as SUP are linked to an elevated risk of violence against close family members (36), these family members often face ongoing and cumulative exposure to such risks within close relationships, highlighting their heightened vulnerability.

Our regression analysis revealed a total reduction in QoL of 0.49 points, indicating that being a partner or parent to someone with SUP is associated with a half-point lower QoL. While this reduction is not extreme on a 10-point scale, it nonetheless represents a meaningful decline. Importantly, our analysis showed that the association between being a partner or parent to someone with SUP and lower QoL was statistically accounted for by exposure to violence. The observed pattern of associations is consistent with the hypothesized mediation model, but cannot confirm it. The findings indicate that exposure to violence is closely associated with lower QoL among family members of individuals with SUP, although other unmeasured factors may also contribute to this association. This finding also aligns with the framework proposed by Orford (56), who, in a large cross-cultural study, found that family members of individuals with SUP experience greater strain and coping difficulties under conditions of accumulated burden. Based on our results, exposure to violence may represent one such burden and appears to be an important factor associated with lower QoL among those exposed to a family member's SUP.

### 4.1 Implications for practice and public health

Given that partners and parents of individuals with SUP experience high levels of violence, our results indicate that professionals working with family members should assess whether they have been exposed to various forms of violence and explore the context in which it occurs. Such an approach may help inform interventions addressing violence in the context of SUP (23). Violence can take various forms and can often be hidden from the outside world for various reasons and might therefore be difficult to detect. Overall, given the strong association between SUP and violence (49), clinical practice should adopt a family-oriented approach that includes screening close family members for experiences of violence (57). Treatment approaches should further adopt an integrated focus on safety, emotion regulation, and coping strategies when family members affected by others' SUP are living with violence, abuse, or other forms of traumatic adversity (58).

Various support groups have been shown to have positive effects for both people with SUP and close family members (27, 50, 58, 59). Clinicians should therefore familiarize themselves with such groups and refer both patients and close family members to them. Meeting others with similar lived experiences may be highly beneficial for affected family members. Support services should provide psychoeducational interventions for affected family members, both individual and group-based formats, and facilitate contact with appropriate peer-led support groups. Such recommendations should also be incorporated into policy and guideline documents.

From a public health perspective, the findings underscore the importance of recognizing affected family members as a potentially vulnerable population and incorporating assessment of violence exposure and support needs into population-based prevention and health promotion initiatives.

### 4.2 Methodological considerations

The study demonstrates a significant strength in its inclusion of a large number of participants representing a diverse age range, who were randomly selected from a general population. However, it is important to acknowledge certain sample biases. Most importantly, the cross-sectional design precludes conclusions about temporal ordering and causal relationships (60). Although violence exposure was assessed for the 5 years preceding the survey, overlap with the measurement of current QoL cannot be excluded. The sample included a higher proportion of individuals with higher education than the general adult population in Norway. Response rates were also lower among young adults, particularly men under 40, indicating potential sampling bias. Despite the relatively lower response rate compared to those typically reported in institution-based surveys, such as those conducted at schools, the response rate was considered acceptable for an online survey (61). Missingness in the violence variables was low overall (~6%), with only minor differences (1–4 percentage points) across gender, SES, and family relation to individuals with SUP (Supplementary Table 1). The slightly higher non-response in some of these groups may have resulted in a slight underestimation of the prevalence of violence. Nonresponse weighting was considered but not applied, as our primary aim was to examine associations rather than produce population-representative prevalence estimates. Although weighting may improve representativeness with respect to sociodemographic characteristics, it was deemed less critical for the analytical objectives of this study. Consequently, prevalence estimates should be interpreted with some caution. Furthermore, although we adjusted for key covariates (age, gender, and financial strain), residual confounding from unmeasured factors that may be associated with reduced QoL—such as caregiving burden, stigma, chronic stress, fear, social isolation, or the respondent's own health or substance use—cannot be excluded.

The findings of the study rely on self-reported data, which may be susceptible to potential underreporting, particularly given the sensitivity of the topics assessed. Relatedly, prevalence estimates should be interpreted with caution due to potential nonresponse bias. While the assumption of missing at random cannot be fully verified, the use of WLSMV allows inclusion of all available data and provides robust parameter estimates under standard assumptions. Recall bias related to sensitive experiences may also have been present. We partly addressed this by limiting the adverse outcomes in adulthood (violence) to the last 5 years.

Regarding measurement and operationalization of key variables, the question identifying respondents as partners or parents of individuals with SUP was combined, preventing us from reporting separate data for each group. Partners and parents may differ with regard to relationship dynamics, caregiving responsibilities, exposure to violence, and psychosocial consequences associated with a family member's SUP. Consequently, combining these groups may obscure important differences between partners and parents, and the findings should therefore be interpreted as representing an overall pattern among close family members rather than effects that necessarily apply equally across these subgroups. The broad, self-defined exposure classification (SUP or substance use disorder) may have introduced heterogeneity in the composition of the group identified as next-of-kin. Furthermore, violence-related questions were asked in general terms and not directly linked to the person with SUP, so we cannot confirm whether the reported violence was specifically related to that family member. Finally, we did not have data on whether the person with SUP was present in the respondent's life during the past 5 years, nor whether the reported violence occurred during periods of contact with that family member. Future research would benefit from distinguishing between relationship types and co-residence to better capture variations in exposure and burden.

## 5 Conclusion

Partners or parents of individuals with SUP have a higher risk of experiencing violence over the last 5 years, compared to those without this family exposure. In gender-stratified analyses, the risk was shown as largely similar for all genders. Findings should be interpreted as cross-sectional associations rather than evidence of causal pathways. In addition, the source of reported violence could not be determined from the available data. Services should be attentive to the challenges these family members face, including exposure to violence, and provide appropriate support.

## Data Availability

Publicly available datasets were analyzed in this study. The dataset that supports the conclusions of this article can be accessed through the Helsedata repository (https://helsedata.no/en/) and can be obtained by submitting an application to the Norwegian Institute of Public Health. For more information, please contact Bente Birkeland.
