# Supplementary material for: Violence exposure and quality of life among partners and parents of individuals with substance use problems
Source: Front Public Health. 2026 Sep 11;14:1863383. doi: 10.3389/fpubh.2026.1863383 (PMC13612343; doi:10.3389/fpubh.2026.1863383)
Supplement: Supplementary file 1 [file Table_1.docx]

**Supplementary Table 1.** Missingness in violence variables by gender, socioeconomic status (SES), and relation to individuals with SUP (N = 17,640)

|  | Physical violence (%) | Sexual violence (%) | Emotional abuse (%) |
| --- | --- | --- | --- |
| Female vs. male | 7 vs 5 | 7 vs 5 | 7 vs 5 |
| Low SES vs high SES | 7 vs 5 | 7 vs 5 | 8 vs 4 |
| Partner or parent of individual with SUP vs. others | 4 vs 6 | 4 vs 6 | 5 vs 7 |
